# Dyclonine rescues frataxin deficiency in animal models and buccal cells of patients with Friedreich's ataxia

**DOI:** 10.1093/hmg/ddu408

**Published:** 2014-08-11

**Authors:** Sunil Sahdeo, Brian D. Scott, Marissa Z. McMackin, Mittal Jasoliya, Brandon Brown, Heike Wulff, Susan L. Perlman, Mark A. Pook, Gino A. Cortopassi

**Affiliations:** 1 Department of Molecular Biosciences, School of Veterinary Medicine; 2 Department of Pharmacology, School of Medicine, University of California, Davis, CA 95616, USA; 3 Department of Neurology, University of California School of Medicine, Los Angeles, CA 90095, USA and; 4 School of Health Sciences and Social Care, Brunel University, UxbridgeUB8 3PH, UK

## Abstract

Inherited deficiency in the mitochondrial protein frataxin (FXN) causes the rare disease Friedreich's ataxia (FA), for which there is no successful treatment. We identified a redox deficiency in FA cells and used this to model the disease. We screened a 1600-compound library to identify existing drugs, which could be of therapeutic benefit. We identified the topical anesthetic dyclonine as protective. Dyclonine increased FXN transcript and FXN protein dose-dependently in FA cells and brains of animal models. Dyclonine also rescued FXN-dependent enzyme deficiencies in the iron–sulfur enzymes, aconitase and succinate dehydrogenase. Dyclonine induces the Nrf2 [nuclear factor (erythroid-derived 2)-like 2] transcription factor, which we show binds an upstream response element in the FXN locus. Additionally, dyclonine also inhibited the activity of histone methyltransferase G9a, known to methylate histone H3K9 to silence FA chromatin. Chronic dosing in a FA mouse model prevented a performance decline in balance beam studies. A human clinical proof-of-concept study was completed in eight FA patients dosed twice daily using a 1% dyclonine rinse for 1 week. Six of the eight patients showed an increase in buccal cell FXN levels, and fold induction was significantly correlated with disease severity. Dyclonine represents a novel therapeutic strategy that can potentially be repurposed for the treatment of FA.

## INTRODUCTION

Friedreich's ataxia (FA) is a severe neurodegenerative disease that is the most common autosomal recessive inherited movement disorder (1). There is no cure or effective treatment for FA. The disease causes degeneration and demyelination in dorsal root ganglion (DRG) neurons and spinocerebellar tracts, resulting in movement and speech disorders (2,3). Symptoms begin between the ages of 5–15 years, and patients are often wheelchair bound within 10–15 years of diagnosis. Early death is common and usually occurs from cardiac complications (4). FA is caused by a decrease in the mitochondrial protein frataxin (FXN gene), which has been shown to have roles in iron–sulfur cluster synthesis, iron transfer and antioxidant defense (1,5–8). The decrease in FXN stems from an accumulation of GAA triplet repeats in the first intron of the gene through inheritance (9–11). Here, we report on screening and identification of a potential therapeutic for FA. We previously identified antioxidant defects in DRG cells of an FA mouse model (12). Using this to develop a specific hypothesis for disease pathogenesis, we designed a cellular disease model for FA in order to identify a potential treatment. The cell model identified dyclonine, and we have shown that it induces FXN in cells, animal models and FA patients through a novel mechanism. Dyclonine is an oral anesthetic used to provide topical anesthesia to mucous membranes through sodium channel inhibition (13). It is the active ingredient in Sucrets, an over-the-counter throat lozenge, and has been in use for over 50 years (14). Additionally, dyclonine is used during dental examinations and procedures to numb mucous membranes of the mouth (15).

## RESULTS

### FXN-deficient cells are sensitive to the thioredoxin-oxidant diamide

**Figure 1.**
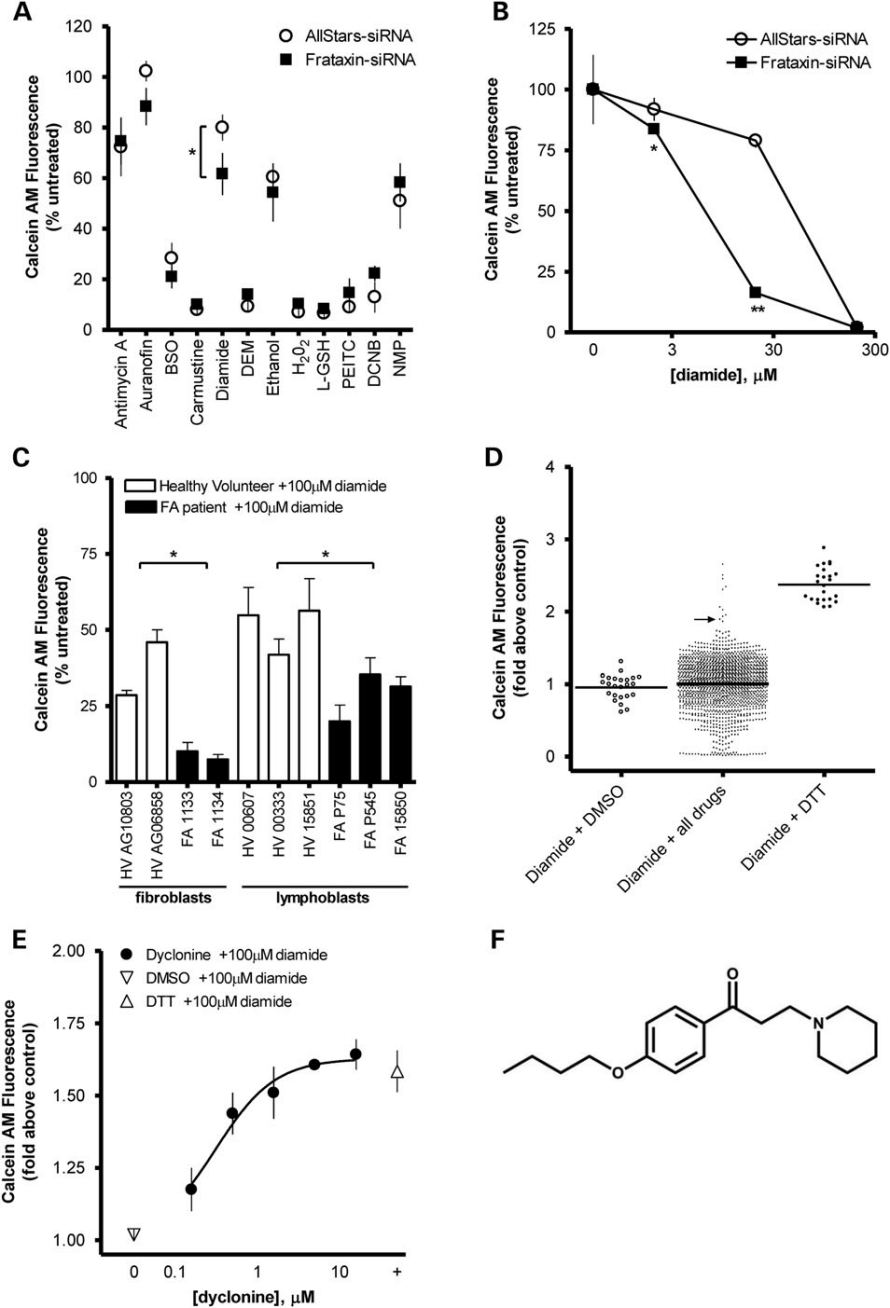
High-throughput screening reveals that dyclonine protects FA patient fibroblasts from diamide stress. (**A**) Effect of antioxidant inhibitors on 50B11 cell viability with FXN knockdown. Eleven inhibitors of thiol-related antioxidants were tested in an siRNA-mediated, FXN-deficient 50B11 DRG cell line [10 µM antimycin A, 1 µM auranofin, 100 µM BSO, 100 µM carmustine, 10 µM diamide, 0.1% diethyl maleate (DEM), 0.1% ethanol, 0.03% H_2_O_2_, 1 mm l-glutathione (l-GSH), 0.1% phenethyl isothiocyanate (PEITC), 100 µM dichloronitrobenzene (DCNB) and 1 µM *N*-methyl protoporphyrin (NMP)]. Cell viability was measured with Calcein-AM after 24 h and normalized to untreated control (*n* = 3). Increased sensitivity to cell death was induced by inhibitors of thiol-related antioxidants diamide and auranofin in FXN knockdown cells compared with AllStars non-targeting siRNA negative control. (**B**) FXN-dependent sensitivity to diamide is dose-dependent in 50B11 cells. Cell viability was measured with Calcein-AM after 24 h of treatment with 3–300 µM diamide and normalized to untreated control (*n* = 3). (**C**) Friedreich's patient cells are sensitive to diamide. To confirm these effects in patient cells with low FXN, we tested 100 µM diamide in fibroblasts and lymphoblasts and found that patient cells were more sensitive to diamide compared with healthy control cells. (**D**) Results of high-throughput screen for drugs that protect from diamide toxicity. Significance is shown comparing healthy volunteer and FA patient lines grouped together. This cell-based assay in FA patient fibroblast cell line 1134 was further optimized for high-throughput screening in 96-well plates, with a mean *Z*′-value of 0.75 (*n* = 25). This platform was used to screen a library of 1600 drugs that have been approved for clinical use. FA fibroblasts were pretreated with 10 µM test compound, DMSO (negative control) or 300 µM dithiothreitol (DTT) (positive control) for 24 h and followed by 100 µM diamide for 24 h. Cell viability was measured with Calcein-AM. Screening data (diamide + all drugs) are the mean of two replicates and presented as fold above DMSO + diamide control. Arrow indicates dyclonine response. Mean + SD for the 1600 drugs was 1 ± 0.3; mean + SEM was 1 ± 0.01. Compounds that rescued from diamide toxicity greater than mean + 2× SD advanced to secondary screening, which included replication of protective effect in a concentration-dependent manner, 0.01–10 µM. (**E**) An example of dose-dependent protection by dyclonine. Dyclonine was added to FA fibroblast line 1134 for 24 h before 100 µM diamide treatment, and Calcein-AM viability is shown as fold above DMSO + diamide control. Intrinsic effect: 2.1 ± 0.49-fold above DMSO + diamide control; EC_50_: 0.36 ± 0.25 μM (*n* = 3). (**F**) Chemical structure of dyclonine. The plotted data in A, B, C and E display mean responses and error bars represent SD (*n* = 3–4). *P < 0.05, **P < 0.001 relative to control, *t-*test.

There is ample support for dysregulated antioxidant defenses in FA (12,16–18). We screened that12 inhibitors of thiol antioxidants were screened in FXN-deficient DRG neural cells, chosen since the DRGs are the primary site of disease pathogenesis (Fig. 1A and B) .Measuring viable cells with Calcein-AM staining, the thioredoxin-oxidant diamide produced the greatest sensitivity (19). To confirm this phenotype in patient cells, we found that FXN-deficient FA fibroblasts and lymphoblasts were also clearly more sensitive to diamide treatment than healthy controls (Fig. 1C). Diamide is a specific thiol oxidant that causes oxidative damage and results in cell death (20,21). In the FA patient fibroblasts, a robust high-throughput screening assay based on diamide sensitivity was developed, with an average *Z*′-score of 0.7. We then screened a 1600-compound library of drugs that have been approved for use in human trials, and identified protective drugs (Fig. 1D). After the initial screen, multiple rounds of hit confirmation, and then concentration response curves, 33 drugs were found that reproducibly protected the patient cells from the thiol stress induced by diamide (Supplementary Material, Table S1). Of the drugs found to protect FA fibroblasts from diamide-induced oxidative stress, dyclonine was one of the most potent (Fig. 1E).

### Dyclonine induces FXN in patient cells and in animal model tissues

**Figure 2.**
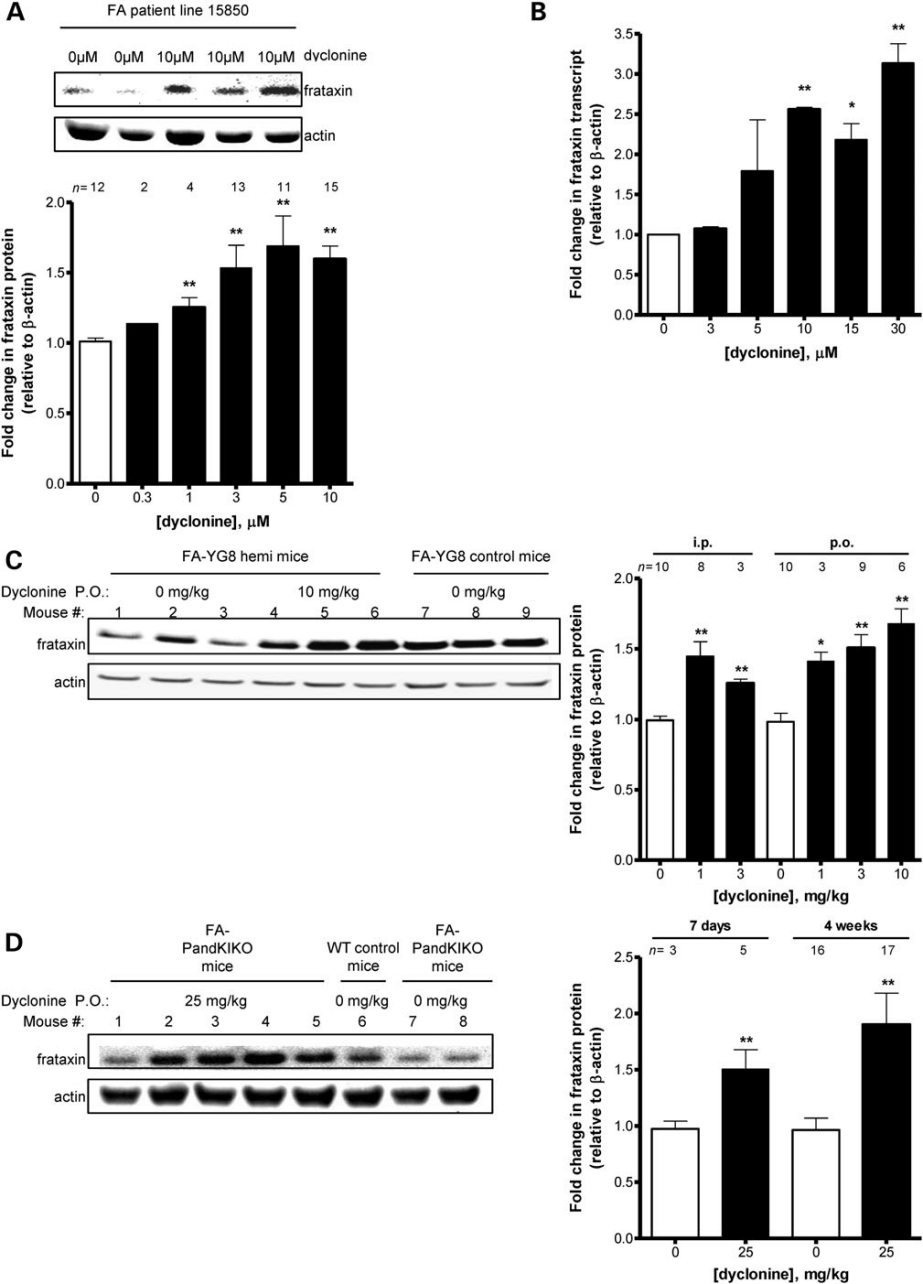
Dyclonine induces FXN in cultured FA patient cells and FA mouse model cerebellum *in vivo*. (**A**) Dyclonine increases FXN protein expression in FA patient lymphoblast cell lines. To test if the mechanism of protection from diamide toxicity for dyclonine was through an increase in FXN protein levels, FA lymphoblasts were treated with 0.3–10 µM dyclonine or vehicle control (0.1% DMSO). Total protein was collected after 48 h, and lysates were probed by western blot analysis for FXN expression and normalized to β-actin. The plotted data represent the mean fold change in FXN protein in drug-treated cells, normalized to vehicle control. Error bars represent SEM. **P < 0.01, *t-*test (*n* = 2–15). A representative blot is shown for FA patient lymphoblast line 15850 treated with 10 µM dyclonine for 48 h. (**B**) Dyclonine increases FXN transcript levels in FA patient lymphoblasts. FA lymphoblast cell line 14518 was treated with 3–30 µM dyclonine or vehicle control (0.1% DMSO). RNA was analyzed after 24 h by RT-PCR with expression with primers for FXN normalized to β-actin. The plotted data represent the mean fold change in FXN transcript in drug-treated cells normalized to vehicle control. Error bars represent SEM. *P < 0.05, **P < 0.01, (*n* = 4), *t-*test. (**C**) Dyclonine increases FXN protein concentration in FA-YG8 mouse cerebellum. To determine the ability of dyclonine to reverse the *in vivo* FXN protein defect, FA-YG8 transgenic mice [*hFXN*^+/−^ with FXN (GAA)_190_ expansion; m*Fxn*^−/−^] were treated with 1–10 mg/kg dyclonine for 1 week i.p. or p.o. Cerebellar lysates were probed by western blot analysis for FXN expression and normalized to β-actin. The plotted data represent the mean fold change in FXN protein in drug-treated mice normalized to vehicle control. Error bars represent SEM. *P < 0.05, **P < 0.01, *t-*test (*n* = 3–10 and 11–12 months of age). A representative blot is shown for cerebellum from FA-YG8 mice treated with 10 mg/kg dyclonine for 1 week. FA-YG8 [*hFXN*^+/+^ with FXN (GAA)_190_ expansion; m*Fxn*^−/−^] mice with two copies of transgene used as a control. (**D**) Dyclonine increases FXN protein levels in FA-PandKIKO mouse cerebellum. To determine the ability of dyclonine to reverse the *in vivo* FXN protein defect in an additional FA model at a higher dose and duration, FA-PandKIKO mice [*mFxn*^+/−^ with FXN (GAA)_230_ expansion; m*Fxn*^−/−^] were treated with 25 mg/kg dyclonine for 1 or 4 weeks p.o. Cerebellar lysates were probed by western blot analysis for FXN expression and normalized to β-actin. The plotted data represent the mean fold change in FXN protein in drug-treated mice normalized to vehicle control. Error bars represent SEM. **P < 0.01, *t-*test. (*n* = 3–17 and 8–10 months of age). A representative blot is shown for cerebellum from FA-YG8 mice treated with 25 mg/kg dyclonine for 1 week. Wild-type C57BL/6 mice used as a control.

Multiple mechanisms of action of protection from diamide were possible. One is reactivation of FXN expression, since its deficiency mediated the sensitivity to diamide. Thus, all 33 confirmed that screening hits including dyclonine were tested for their ability to induce FXN protein levels in the absence of diamide (Supplementary Material, Table S1). Of the compounds found to increase FXN in FA patient cells, we eliminated some because of safety concerns (i.e. cotinine and nifursol), and others because of their primary use is in veterinary medicine (i.e. oxfendazole) (22–24). We chose to follow-up with dyclonine because of its potency of diamide protection, reproducibility of FXN induction and safe use in humans for decades. FA patient lymphoblasts treated with dyclonine show clear induction of FXN protein after 48 h of dyclonine exposure (Fig. 2A). Additionally, FXN mRNA transcript measured by RT-PCR is also dose-dependently increased after 24 h drug treatment in FA lymphoblasts (Fig. 2B). We observed induction of FXN in multiple animal models after intraperitoneal or oral dosing. FA-YG8 [*hFXN*^+/−^ with FXN (GAA)_190_ expansion; m*Fxn*^−/−^] transgenic mice (8) dosed for 1 week once daily with dyclonine i.p. or p.o. showed a clear induction of FXN protein in cerebellar lysate (Fig. 2C).

In addition, to explore the effects of dyclonine in an additional model with chronic dosing, we treated FA-PandKIKO mice [*mFxn*^+/−^ with FXN (GAA)_230_ expansion; m*Fxn*^−/−^] with 25 mg/kg dyclonine for 4 weeks p.o*.* This higher dose was chosen in an attempt to ascertain the maximum induction of FXN within the limits of dyclonine solubility. Thus, showing dyclonine can reverse the *in vivo* FXN protein defect in an additional FA model at a higher dose and chronic duration (Fig. 2D). These chronic studies resulted in no observable changes in sedation, feeding behavior or gross pathology. We observed these effects on FXN in multiple additional tissues, including heart and liver (Supplementary Material, Fig. S1).

### Dyclonine activates the Nrf2 pathway

**Figure 3.**
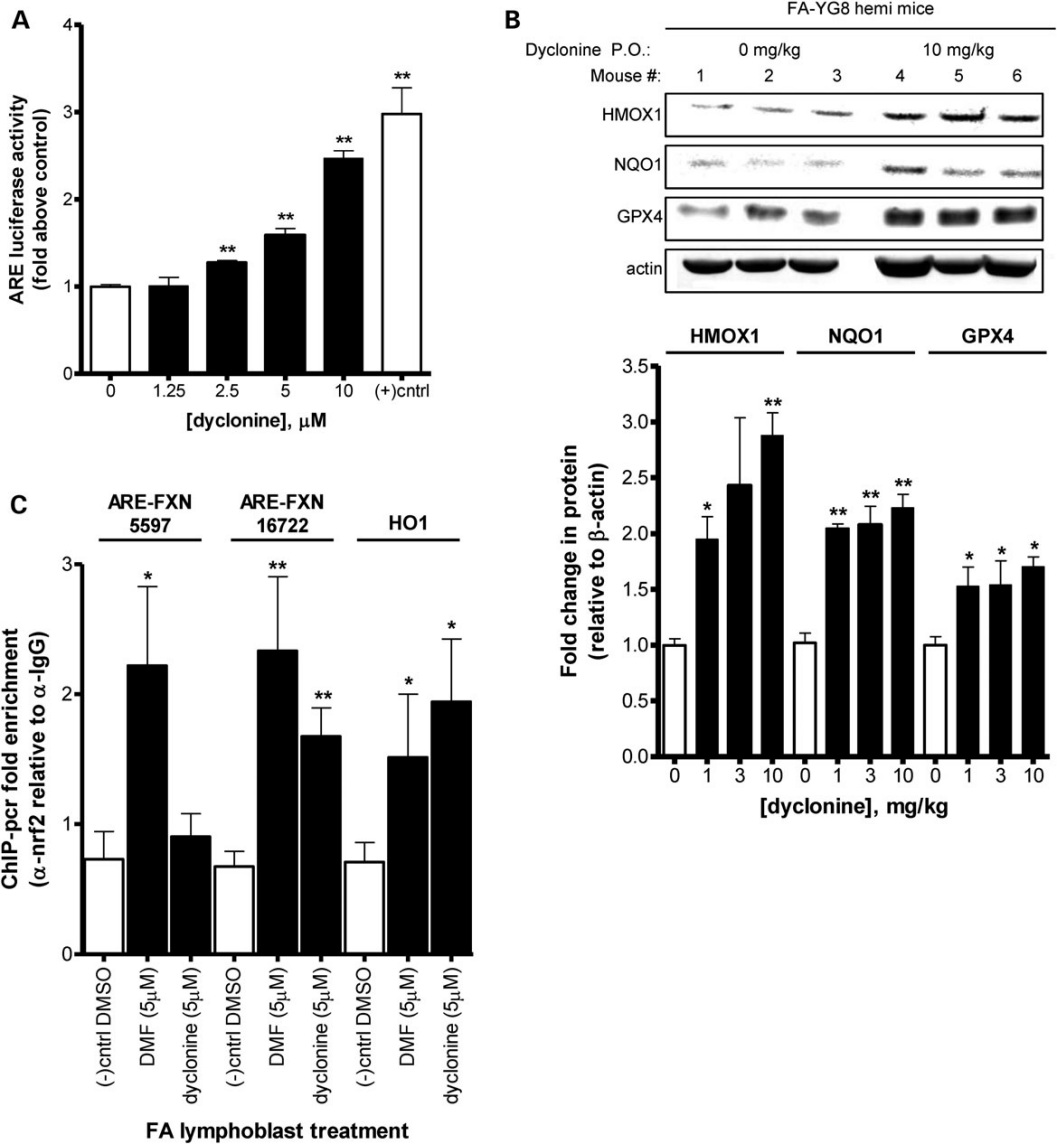
Dyclonine drives an induction of Nrf2 through the AREs in FXN gene. (**A**) Dyclonine increases the expression of ARE-luciferase reporter gene. To explore the mechanism of FXN induction by dyclonine, effects on the nrf2-target ARE were evaluated in a reporter HeLa cell line transduced with ARE-luciferase. Cells were treated with 1.25–10 µM dyclonine or vehicle control (0.1% DMSO). After 24 h, cells were lysed and luciferase activity was measured on a plate reader. The plotted data represent the mean fold change in luminescence in drug-treated cells normalized to vehicle control. (+) control = 5 µM sulforaphane. Error bars represent SEM. **P < 0.01, *t-*test (*n* = 3). (**B**) Dyclonine increases nrf2-target protein expression in the FA mouse cerebellum. FA-YG8 transgenic mice [*hFXN*^+/−^ with FXN (GAA)_190_ expansion; m*Fxn*^−/−^] were treated with 1–10 mg/kg dyclonine for 1 week p.o*.* Cerebellar lysates were probed by western blot analysis for nrf2-target proteins HO1, NQO1, and GPX4 expression and normalized to β-actin. The plotted data represent the mean fold change in FXN protein in drug-treated mice normalized to vehicle control. Error bars represent SEM. *P < 0.05, **P < 0.01, *t-*test. (*n* = 3). A representative blot is shown for cerebellum from FA-YG8 mice treated with 10 mg/kg dyclonine for 1 week. (**C**) Dyclonine induces nrf2 binding to ARE sites in *Fxn* and *Hmox1*. Multiple ARE sites were found upstream of FXN gene (Supplementary Material, Fig. S2). The top two candidates 5597 and 16722 bp upstream were selected from position weight. ChIP assays were performed to determine the binding of Nrf2 to these sites and the promoter of *Hmox1* as a positive control. FA lymphoblasts (GM14518, GM15850 and GM16220) were treated with vehicle (0.1% DMSO), 5 µM dyclonine or 5 µM of the nrf2-inducer dimethyl-fumarate for 24 h. Chromatin was immunoprecipitated with anti-nrf2 antibody or anti-IgG negative control and by PCR for target loci. The plotted data represent the mean fold enrichment of PCR product with Nrf2 pulldown compared with IgG control. This shows increased amplification of regions of DNA for both the ARE sites, 16 722 bp upstream of *fxn* and *Hmox1* after dyclonine treatment. Error bars represent SEM. *P < 0.05, **P < 0.01, *t-*test (*n* = 5 individual pulldowns per condition, *n* = 3 per experiments).

Of the 33 drugs that protected FA patient cells from diamide stress, some drugs were reported antioxidant response element (ARE)/nuclear factor (erythroid-derived 2)-like 2 (Nrf2) inducers in literature such as ebselen (25). Nrf2 is a transcription factor that responds to oxidative and thiol stress by binding AREs and driving the expression of multiple antioxidant and anti-inflammatory target genes including heme oxygenase 1 (HMOX1), NAD(P)H dehydrogenase (quinone) (NQO1) and glutathione peroxidase (GPX4) (26,27). We explored the activity of all 33 drugs as ARE/Nrf2 inducers for their ability to stimulate ARE-luciferase reporter system activity (Supplementary Material, Table S1), including dyclonine which dose-dependently drove induction of ARE-luciferase (Fig. 3A). Remarkably, multiple drugs that increased ARE-luciferase expression were also found to be FXN inducers (Supplementary Material, Table S1). In addition, after oral dyclonine dosing in the FA-YG8 mouse model of FA drives a dose-dependent increase in known Nrf2-target proteins in cerebellar lysate including heme oxygenase (HO1), NQO1 and GPX4 (Fig. 3B). Thus, dyclonine is an activator of the ARE/Nrf2 pathway.

### Evolutionarily conserved Nrf2-binding sites (AREs) exist in the FXN gene and are functional Nrf2-binding sites

We studied the mechanism of induction of FXN by dyclonine with regard to Nrf2. If dyclonine induces FXN through the Nrf2 pathway, then there should be active Nrf2-binding sites, i.e. AREs (28) within the FXN locus that are triggered by dyclonine exposure. Seven potential ARE sites were found between 20 kb upstream and 5 kb downstream of the FXN locus (Table 1). The top three potential ARE sites had excellent ARE scores of 12.9, 12.5 and 8.0, and were found 4.9, 5.6 and 16.7 kb, respectively, upstream of the transcription start site for FXN (Supplementary Material, Fig. S2).

**Table 1.** ARE sites in human FXN gene

Position	Position relative to FXN	Direction	DNA strand	Sequence	PWM score	Evolutionarily conserved (95% CI)
71633757	−16 722	Antisense	Non-template	ACCATGTGACAATGCCCACTT	7.80	Yes
71634506	−15 973	Sense	Template	CCTGGGTGACAGAGCAAGACT	12.30	No
71636687	−13 792	Antisense	Non-template	AGGAACTGACTCCGCACAAGA	10.30	No
71639782	−10 697	Antisense	Non-template	AGTAGATGACCCAGCTGATAG	8.90	No
71643186	−7293	Sense	Template	ACAGTGTGACTATGCTCGACT	10.20	No
71644882	−5597	Antisense	Non-template	GACTCATGACTCAGCCAGTCC	12.50	Yes
71645605	−4874	Antisense	Template	GACCGGTGACTTTGCAAGTTA	12.90	Yes
71651446	967	Antisense	Template	AACCTTTGACTGGGCTGGAAA	4.20	No
71654599	4120	Sense	Template	GGGATGTGACGGGGCTGCGTC	1.20	No

FXN gene starts at 71650479 and ends at 71715094 (64 615 bp). GAA-repeat insertion is 1731 bp downstream of FXN exon-1 start site. Position weight matrix (PWM) score from Wang *et al*. (28). Searched for the regular expression of ARE consensus sequence: TGAC[ACTG]{3}GC from 20 kb upstream to 5 kb downstream of FXN.

All potential ARE sites were compared among the FXN loci of the great apes aligned with Clustal W (Homo sapiens, Pan troglodytes, Gorilla and Pongo abeli) in the 10 kb region upstream of the FXN gene (29). The top three canonical ARE sites were perfectly conserved at all 21 bp among all four species. Chromatin Immunoprecipitation (ChIP) experiments were carried out in drug-treated FA patient lymphoblasts, using an antibody to pull down Nrf2 and PCR using primers to the potential ARE sites in FXN or control Nrf2-target genes. These demonstrate that dyclonine enriches binding of Nrf2 to the ARE site in HO1 and the FXN gene 16 722 bp upstream of the start site (Fig. 3C). Thus, there are active ARE sites in the FXN gene.

### Epigenetic effects of dyclonine on the FXN locus

**Figure 4.**
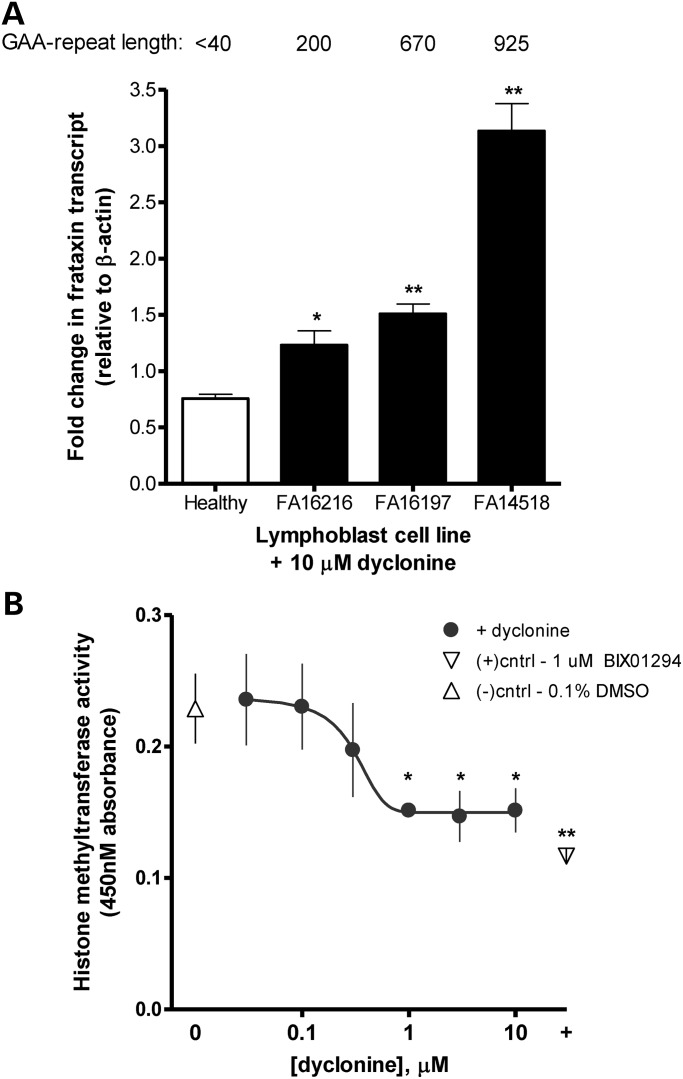
Dyclonine exerts epigenetic effects. (**A**) Dyclonine induction of FXN transcript levels in healthy and FA patient lymphoblast cells with varying GAA-repeat length. Healthy and FA patient lymphoblasts (GM16216, GM16197 and GM14518) were treated with 30 µM dyclonine or vehicle control (0.1% DMSO). RNA was analyzed after 24 h by RT-PCR with expression with primers for FXN normalized to β-actin. GAA, number of GAA repeats on FXN allele with less insertions. The plotted data represent the mean fold change in FXN transcript in drug-treated cells normalized to vehicle control for that cell line. Error bars represent SEM. *P < 0.05, **P < 0.01, *t-*test (*n* = 4). The induction of *Fxn* mRNA increases with GAA repeat length. (**B**) Dyclonine inhibits histone methyltransferase activity. Histone methyltransferase G9a (G9aHMTase) activity, which specifically methylates histone H3K9, was measured in nuclear extracts of healthy lymphoblast cells treated with drug for 60 min. Negative controls are 0.1% DMSO vehicle-treated cells; positive controls are cells treated with G9aHMTase-specific inhibitor 1 µM Bix01294. The plotted data represent the mean absorbance at 450 nm which reflects G9aHMTase activity. Error bars represent SEM. *P < 0.05, **P < 0.01, *t-*test. (*n* = 4).

In FA, inheritance of larger GAA expansions causes increased epigenetic silencing, decreased FXN expression, and increased disease severity (6,30). The mechanism of dyclonine-induced FXN re-expression was investigated in patient lymphoblast cells bearing differential GAA repeats (Fig. 4A). The ability of dyclonine to induce FXN expression is directly correlated with dose and repeat length, in that larger, expanded, more silenced FXN loci were more ‘reactivatable’ by dyclonine.

Thus, as a result of Nrf2 activation or as a cause of it, dyclonine has an ‘unsilencing’ epigenetic activity. Dyclonine has been reported to have inhibitory activity on histone lysine methyltransferase G9a, which is known to methylate Histone H3K9 (PubChem, assay ID 504332). Inhibitory methylation of histone H3K9 is a known epigenetic mark at the FXN locus (31–34). We found that dyclonine inhibited histone methyltransferase G9 in lymphoblast nuclear extracts (Fig. 4B). Recently, sulforaphane's activation of Nrf2 has been demonstrated to occur through an epigenetic unsilencing of the Nrf2 locus itself (35,36). Thus, either dyclonine → G9A → FXN and Nrf2, or dyclonine → Nrf2 → FXN, are possible.

### Dyclonine inhibits sodium channels, but does not produce systemic anesthesia

The mechanism of dyclonine's well-known anesthetic actions is through inhibition of sodium channels (37), though the concentrations were not readily available in literature. We tested the inhibitory effect of dyclonine using patch-clamp electrophysiology on sodium channel NaV1.2 currents, because of its presence on neurons and potential for sedative effects (38,39). As expected, we found that dyclonine clearly inhibited the sodium channel current in N1E–115 neuroblastoma cells. (Supplementary Material, Fig. S3). Since dyclonine is used primarily as a topical anesthetic (13), we tested whether dyclonine induced systemic anesthesia when dosed systemically at doses relevant for FXN induction. We measured hot plate response times in mice dosed with 3 mg/kg dyclonine i.p., and observed no delay in hot plate response over 1.5 h (Supplementary Material, Fig. S4), meaning dyclonine does not produce systemic anesthesia at these doses.

### Rescue of biochemical and behavioral end points of FXN deficiency by dyclonine

**Figure 5.**
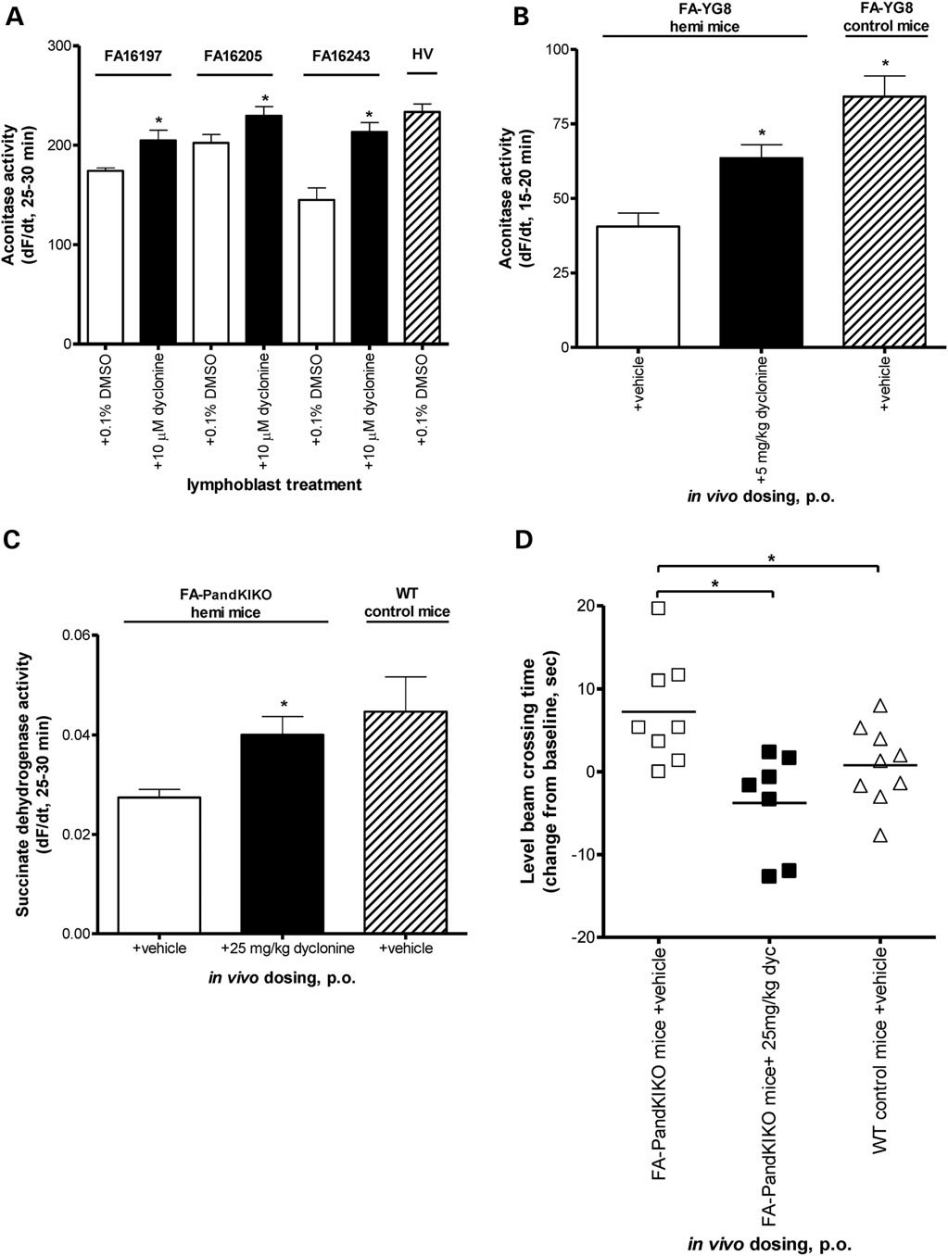
Dyclonine recovers downstream effects of FXN deficiency *in vitro* and *in vivo*. (**A**) Dyclonine increases aconitase activity in FA patient lymphoblast cell lines. FA lymphoblasts (GM16205, GM16197 and GM16243) were treated with 10 µM dyclonine or vehicle control (0.1% DMSO). Cell pellets were collected after 48 h, and aconitase activity was measured in lysates over 90 min to ensure linear range was captured. A healthy volunteer lymphoblast cell line was used as a control. The plotted data represent the mean rate change in fluorescence (d*F*/d*t*) from 25 to 30 min. Error bars represent SEM. *P < 0.05, *t-*test. (*n* = 3). (**B**) Dyclonine increases aconitase activity in FA mouse model cerebellum *in vivo*. FA-YG8 transgenic mice [*hFXN*^+/−^ with FXN (GAA)_190_ expansion; m*Fxn*^−/−^] were treated with 5 mg/kg dyclonine for 1 week p.o*.* Aconitase activity was measured in cerebellar lysates over 90 min to ensure linear range was captured. Positive controls were vehicle-treated FA-YG8 transgenic mice [*hFXN*^+/+^ with FXN (GAA)_190_ expansion; m*Fxn*^−/−^]. The plotted data represent the mean rate change in fluorescence (d*F*/d*t*) from 15 to 20 min. Error bars represent SEM. *P < 0.05, *t-*test (*n* = 4 mice per group, 11–12 months of age). (**C**) Dyclonine increases succinate dehydrogenase activity in FA mouse model liver *in vivo*. FA-PandKIKO mice [*mFxn*^+/−^ with FXN (GAA)_230_ expansion; m*Fxn*^−/−^] were treated with 25 mg/kg dyclonine for 1 week p.o*.* Succinate dehydrogenase activity was measured in liver lysates over 90 min. Positive controls were vehicle-treated WT C57/Bl6 mice. The plotted data represent the mean rate change in fluorescence (d*F*/d*t*) from 25 to 30 min. Error bars represent SEM. *P < 0.05, *t-*test (*n* = 4 mice per group, 8–9 months of age). (**D**) Dyclonine improves progressive behavioral defect of FA mice. FA-PandKIKO mice [*mFxn*^+/−^ with FXN (GAA)_230_ expansion; m*Fxn*^−/−^] showed a pronounced increase in beam crossing time compared with WT controls at 13 months of age. FA-PandKIKO mice were treated with 25 mg/kg dyclonine or vehicle for 4 weeks p.o*.* Latency to cross a 16 mm beam was the recorded time from start to finish of the beam, with the experimenter blinded to treatment groups. The plotted data represent the mean latency to cross a beam post-dosing minus the time to cross pre-dosing for each individual mouse and serve as a change from baseline measurement. As a change from baseline, a negative number means that the animal was faster after dosing than it was before dosing. Positive controls were vehicle-treated WT C57/Bl6 mice. *P < 0.05, *t-*test (*n* = 7–9 mice per group, 12–14 months of age).

FXN plays a functional role in iron–sulfur cluster biogenesis (40,41), and FXN knockdown of patient cells causes defects in the activity of the iron–sulfur cluster-containing enzymes, aconitase and succinate dehydrogenase (42,43). We observed induction of aconitase activity in patient lymphoblast extracts after dyclonine treatment for 24 h in three FA patient lymphoblast lines (Fig. 5A). Also, dyclonine treatment for 1 week increased FA-YG8 mouse cerebellar aconitase activity (Fig. 5B). In addition to aconitase, succinate dehydrogenase activity was measured in mouse liver and daily oral dyclonine treatment for 4 weeks reversed the succinate dehydrogenase defect in mouse liver lysate (Fig. 5C). Additionally, our behavioral analysis of FA-PandKIKO mice demonstrates that 16 mm level beam crossing time is the most reproducible measure in these FXN-deficient mice. After 4 weeks of oral dosing with 25 mg/kg dyclonine, vehicle-dosed FA mouse balance beam time worsened by on average 8 s, whereas dyclonine-dosed animals not only did not worsen, but improved their times across (Fig. 5D and Supplementary Material, Fig. S6). Additionally, analysis of video recordings of mouse beam performance revealed improvement in errors (footslips) after treatment with dyclonine (Supplementary Material, Fig. S6). There were no significant changes in body weight or feeding behaviors (Supplementary Material, Table S2)

### Oral dosing with dyclonine rinse solution in humans induces FXN in buccal cells

**Figure 6.**
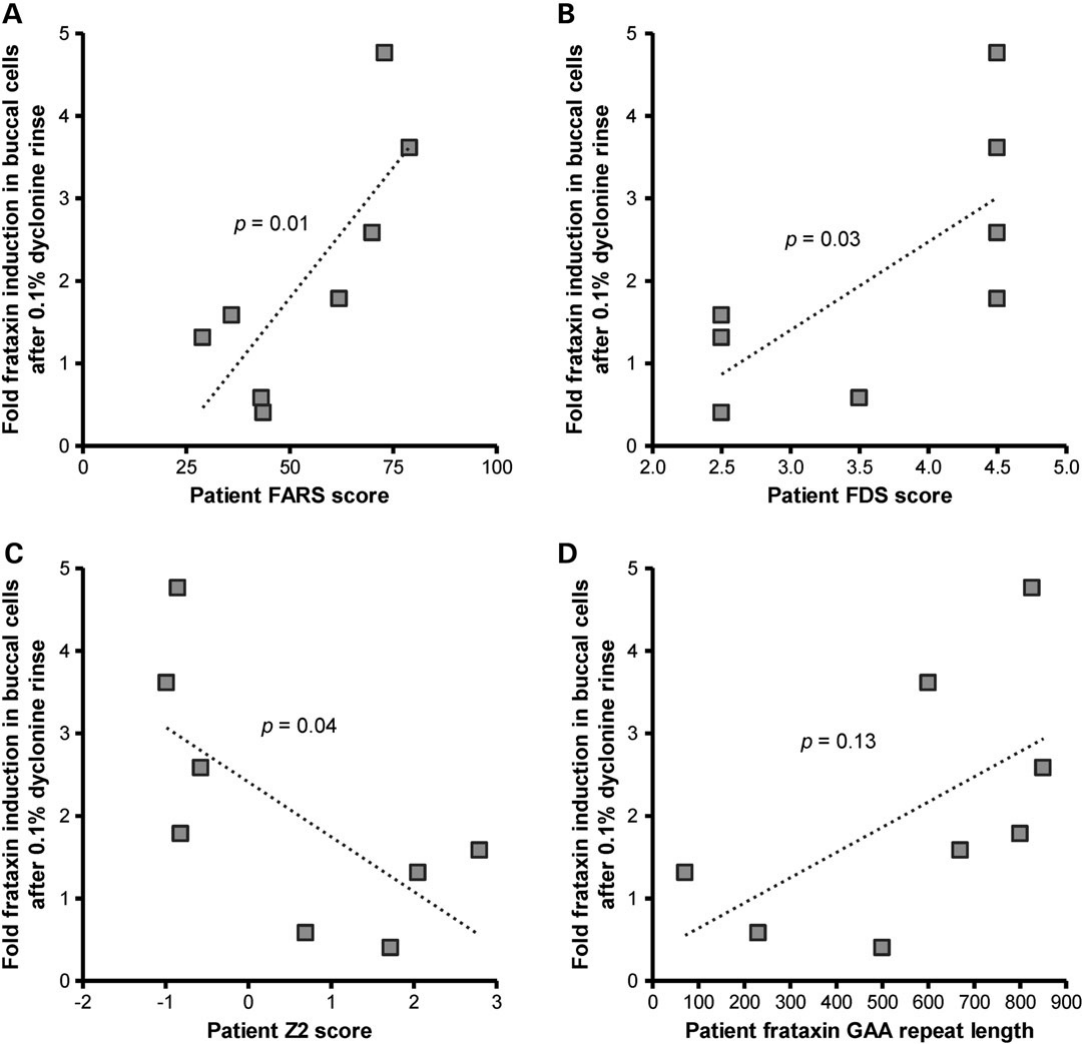
Dyclonine induction of FXN in buccal cells of FA patients correlates with disease severity. FA patients were dosed with a 1% dyclonine oral rinse, twice daily for 30 s. Buccal cells were collected by cheek swabbing before and after 1 week of dosing. Cell lysates were probed by dipstick immunoassay for analysis of FXN expression and normalized to mitochondrial complex IV (*n* = 4 replicate experiments of FXN analysis per patient, summarized in Table 1). The plotted data represent the mean fold change in FXN protein in dyclonine-treated FA patient buccal cells normalized to expression for each individual patient before drug dosing (healthy volunteers not shown). This fold FXN induction is plotted against (**A**) FARS score, (**B**) FDS, (**C**) Z2 ataxia functional composite score and (**D**) FXN gene GAA-repeat length for each FA patient who participated in this clinical study (obtained through a natural history database). For A and B, a higher score indicates greater disease severity. For C, a lower score indicates greater disease severity. A *P*-value of <0.05 is considered significant, Pearson's correlation coefficient.

Since dyclonine is FDA-approved as an oral rinse, and FXN expression can be investigated non-invasively in oral mucosa cells after collection by cheek swab (44,45). Thus, an investigator-initiated clinical study was carried out to investigate the effect of dyclonine on cells in FA patients. Patients came in for initial pre-dosing swab to establish baseline and to receive dyclonine. Patients or caregivers dosed with dyclonine by an oral rinse for 6–7 days twice daily, and then came in for a final cheek swabbing for buccal cells. These blinded samples were then sent for analysis. FXN protein was analyzed by the clinically validated dipstick immunoassay (44,45). We modified these methods by further by normalizing FXN to mitochondrial cytochrome oxidase expression, i.e. FXN/Complex IV (CIV). Four repeats of the dipstick immunoassay were performed per patient sample. Of the 8 patients who received dyclonine, six experienced FXN increase (ranging from 60 to 480% induction), and two experienced no induction (Table 2). Interestingly, patients with greater neurological impairment as measured by the Friedreich's ataxia rating scale (FARS) (46,47), experienced greater FXN induction (Fig. 6A). Other correlations that were also significant included the Functional Disability Score (FDS) and Z2 ataxia functional composite score (Fig. 6B and C) (48). While the correlation of FXN response with GAA repeat length was not significant (Supplementary Material, Fig. 6D), this trend could underlay the differential response seen in Figure 4A.

**Table 2.** Buccal FXN expression in FA patients after treatment with a 1% dyclonine oral rinse

Patient	GAA1	GAA2	Fold induction	*P*-value
FRDA4522	600	500	0.4 ± 0.1	0.01
FRDA0055	1178	826	4.8 ± 1.9	0.01
FRDA0171	600	600	3.6 ± 1.7	0.02
FRDA0157	850	850	2.6 ± 1.4	0.01
FRDA0235	1122	230	0.6 ± 0.3	0.02
FRDA4539	1045	670	1.6 ± 0.5	0.07
FRDA4579	1133	71	1.3 ± 0.6	0.31
FRDA4576	800	800	1.8 ± 0.6	0.04
HV0001	ND	ND	5.2 ± 1.9	0.02
HV0002	ND	ND	1.7 ± 0.3	0.01

Patients and controls were dosed for 1 week with a 1% dyclonine oral rinse, twice daily for 30 s. Buccal cell samples were taken by clinician use swabs and blinded prior to analysis. GAA1 and GAA2 refer to the number of GAA repeats in both alleles of the patient's FXN gene (ND, not determined). FXN was measured in blinded samples using dipstick immunoassay normalized to complex IV expression. Fold induction is a value post-treatment divided by pre-treatment FXN/CIV ± SD (*n* = 4 measurements per patient).

*P*-value determined by the *t*-test.

## DISCUSSION

FA is caused by inheritance of (GAA)*_n_* expansions in intron 1 of the nuclear FXN gene that decreases its expression to about 20% of normal. All consequences of Friedreich's are thought to result from this epigenetic silencing of FXN (5,6,11,49). There is currently no approved or effective therapy for this ultimately lethal disease that currently affects approximately 6000 in the USA and 20 000 in Europe (1). We identified a biochemical deficiency in Friedreich's patient cells to design and perform a drug screen, and have identified a potential treatment for the disease that rescues FXN deficiency as well as downstream consequences in cells, animal models and in FA patients.

Our previous microarray of Friedreich's mouse model DRGs indicated defects in thioredoxin reductase, thiol-antioxidant transcripts and a deficiency in Nrf2 activity in FXN knockdown cells (12). Also, a deficiency in Nrf2 activation was noted in Friedreich's fibroblasts (18). Taken together, these data suggested that FXN deficiency may cause deficiency in Nrf2 activation or the related mitochondrial thioredoxin reductase pathway (50), which leads to decreased mitochondrial antioxidant protection, increased reactive oxygen species, inflammation and neurodegeneration (51).

Given the data on thiol antioxidant deficiency in mouse DRG (12), and previous data on antioxidant deficiency in FA fibroblasts (12,16,17), we tested poisons of thiol antioxidants in a FXN-deficient DRG cell line (Fig. 1A). In these cells, diamide and auranofin were more toxic to FXN knockdown cells compared with controls, whereas hydrogen peroxide (H_2_O_2_) and buthionine sulfoxamine (BSO) displayed little to no difference. This was interesting because diamide oxidizes thioredoxin (52), and auranofin is an inhibitor of thioredoxin reductase (53). These data support the idea that, in the context of the dorsal root ganglia, thioredoxin reductase is an important antioxidant system. Others have shown that, in neural mitochondria, thioredoxin reductase is the dominant antioxidant system (54,55). We examined FA patient cells and also observed increased cell death after diamide treatment which was reversed by the reductant dithiothreitol (DTT), confirming a thiol-oxidant mechanism (Fig. 1C).

To expedite the search for clinical candidates, we took a repurposing approach and examined a collection of 1600 drugs with mostly known pharmacokinetic and safety profiles. Our initial screen identified 100 protective hits in a range of drug classes, of which 33 were found to be reproducibly dose-dependent, for a hit rate of ∼2.5% (Supplementary Material, Table S1). One of the strongest hit in terms of efficacy and potency at protecting from diamide stress was dyclonine, the active agent in Sucrets. Dyclonine was initially characterized as an anti-epileptic (56), but through its sodium channel inhibitory properties it has been used for anesthesia since 1955 (13). Dyclonine's protective effect could potentially be due to multiple mechanisms, including increased mitochondrial biogenesis, Fe/S cluster biogenesis, iron binding or others. Since all FA consequences are the result of deficient FXN expression, we explored whether dyclonine was able to induce FXN in FA patient cells and the animal model (Fig. 2). We discovered an induction of FXN protein, and this was supported further with dose-dependent increases in FXN mRNA by RT-PCR. This has implications for therapy, since dyclonine rescues the protein deficiency that causes the disease in FA lymphoblasts and fibroblasts. In animal models of FA, we observed a clear 2-fold induction in FXN protein in two mouse models, in multiple tissues including cerebellum, liver and heart (Fig. 2C and D, and Supplementary Material, Fig. S1), two of which (cerebellum and heart) are major sites of disease pathogenesis. Our animal studies included intraperitoneal and oral dosing, with drug exposures from 4 days to 4 weeks. In all, we have done 12 independent *in vivo* studies with dyclonine with between 3 and 6 mice per group, and each study supported an induction in FXN by dyclonine. Since FA patients are asymptomatic when their FXN levels are 40–50% of normal levels, an induction of 2-fold should boost patient FXN levels into this range, and alleviate consequences of FXN deficiency (57).

Early on in our development efforts, we noted that a few of the drugs we found to protect from diamide were also Nrf2 inducers in literature. Nrf2 translocates to the nucleus in response to oxidative stress (and especially thiol stress) and binds to AREs in target genes (27). Dyclonine had never been described to induce Nrf2, and we demonstrated a dose-dependent stimulation, and that multiple Nrf2-target proteins are elevated after treatment with dyclonine in both FA patient cells and animal model tissues (Fig. 3B). To investigate if the induction of FXN was Nrf2-mediated, we used bioinformatics to identify ARE sequences in the FXN locus, finding three ARE sites upstream of the transcription start site of FXN that are completely conserved across ape evolution (Table 1 and Supplementary Material, Fig. S2). The functionality of ARE sites was verified by ChIP and pull down with an Nrf2 antibody (Fig. 3C). Thus the induction of FXN is likely the result of recruitment of Nrf2 to adjacent ARE sites. Data also suggest that other Nrf2 inducers will reactivate FXN expression.

We observe that dyclonine has a differential effect on FXN re-expression from alleles of different expansion length. Specifically, longer GAA alleles, which experience greater epigenetic silencing, are more ‘reactivatable’ by dyclonine in cells (Fig. 4A), and this is consistent with what is observed in patients (Fig. 6C). There is precedent for Nrf2 activation having epigenetic effects on genes (35,58–60). There is also recent precedent that the classical Nrf2-inducer sulforaphane may be working through epigenetic unsilencing of the Nrf2 locus (35,36). Dyclonine has been reported to have inhibitory activity on histone lysine methyltransferase G9a, which is known to methylate histone H3K9 (PubChem, assay ID 504332). Inhibitory methylation of histone H3K9 is a known epigenetic mark at the FXN locus (31–34). And we observed experimentally that dyclonine inhibited histone methyltransferase G9 in lymphoblast nuclear extracts (Fig. 4B). These data support either of two mechanisms, i.e. that dyclonine induces Nrf2, and Nrf2 through its epigenetic unsilencing effects reactivates FXN; or that dyclonine suppresses histone methyltransferase G9, which in turn reactivates both the FXN locus and the Nrf2 locus.

Since dyclonine is an anesthetic whose pain ameliorating effects are mediated through sodium channel inhibition, there could be concern of the sedative potential of inhibiting neuronal NaV1.2 sodium channels (61). Additionally, sodium channel blockers are used to treat cardiac arrhythmias and alteration of heart rate would be a concern prior to treatment FA patients with systemic dyclonine. Since there were no data in the literature about the specific concentrations at which this occurred, we examined inhibition of NaV1.2 currents using patch-clamp electrophysiology. As expected, there was clear dose-dependent inhibition with an IC_50_ of ∼6 µM. This IC_50_ is 30-fold higher than our EC_50_ for protection from diamide in FA fibroblasts, and we observe FXN induction in FA lymphoblasts with 1 µM dyclonine. Additionally, after 4 weeks of chronic daily dosing in mice, no sedation or adverse phenotype was observed.

To verify that there was no systemic peripheral anesthesia from ingestion of a topical anesthetic at the doses that induce FXN reactivation, we measured hot plate response and found no delay. Finally, we tested ∼20 sodium channel inhibitors or anesthetics including lidocaine, bupivicaine and propofol and none were significantly active in the diamide protection assay. Taken together, this reduces the concern that the protective actions of dyclonine are mediated through sodium channel blockade, since the doses required for FXN activation are lower than those for sodium channel engagement.

The most well-defined downstream function for mammalian FXN is in iron–sulfur cluster biogenesis (40,42,62), and dyclonine treatment induced iron–sulfur cluster enzyme activity in animal and cell models (Fig. 5A–C). We also examined several neurobehavioral parameters in multiple FA mouse models, including open field activity, grip strength, treadscan and motor coordination on the level beam task. The most sensitive behavioral test was time to cross a level beam. Cerebellar defects in FA patients lead to motor coordination difficulties, and the level beam test mimics the walking score in the FARS ataxia score (47). We observed a significant improvement in this parameter after 4 weeks of oral dyclonine dosing.

Since dyclonine is FDA-approved as an oral rinse, its prescription and dispensing was allowed in an IRB-approved trial. Ten patients were recruited, but 2 did not complete the treatment and stopped 2–3 days into the study, likely because of the taste or mouth numbing that persisted for up to 1 h after each dosing. Of the remaining patients, 6 of the 8 showed an increase in FXN after 1 week. Additionally, both healthy volunteers responded to dyclonine with an increase in FXN (Table 2). The correlations with neurological scores are highly significant, and suggest that dyclonine produces a greater induction in patients with more severe FXN deficiency (Fig. 6).

FXN deficiency causes oxidative stress and subsequent cell death in dorsal root ganglia and cardiac tissue (3). Oxidative stress is suspected to be important in multiple neurodegenerative diseases (63–65). Here, we describe a well-known anesthetic drug dyclonine that protects FA patient cells from diamide-induced oxidative stress, increases FXN and rescues primary biochemical end points of the disease in cell and animal models, and appears to do so involving the Nrf2/ARE pathway. Limitations of this work include translation of beneficial effects seen in mice to humans, as well as the hope that FXN engagement after topical exposure with a rinse solution of dyclonine will model results after a systemic dosing regimen in FA patients. Although there are a number of questions that need to be addressed for these findings to be clinically applied to patients, including a better understanding of pharmacokinetics, optimal dosing formulation for oral bioavailability and cardiovascular toxicology, dyclonine represents a potential novel treatment strategy for the disease.

## MATERIALS AND METHODS

### Cell lines

Human control and FA patient fibroblasts and lymphoblasts were obtained from Coriell Institute for Medical Research repository. Patient lymphoblast cell lines used: GM16214, GM04079, GM16205, GM16243, GM15850, GM16220 and GM16197. Patient fibroblast lines used: 1133, 1134.

### Diamide screening assay

Patient fibroblasts were grown in MEM media (Life Technologies Corp.) supplemented with 15% fetal bovine serum in t225 tissue culture flasks and kept below 70% confluency. Cells were trypsinized and density determined using a Vi-Cell counter (Beckman Coulter Corp). Five thousand fibroblast cells were then aliquoted into 96-well poly-d-lysine-coated black/clear culture plates (Becton Dickinson Corp.) in growth media without antibiotics in a volume of 180 µl. Cells were allowed to adhere for 3–4 h at 37°C. Drugs (10 mm stock in dimethyl sulfoxide, DMSO) were dispensed into assay plate wells after an intermediate dilution in PBS, giving a final DMSO concentration of 0.1% using an electronic multichannel pipetter (BioHit, Sartorius Corp.). Pharmakon drug library consisted of 1600 compounds (Microsource Discovery Systems, Inc.) in 96 well plates at stock concentration of 10 mm in DMSO. Test compounds were all be tested at 10 µM final assay concentration in primary screen. Eight wells each of 300 µM dithiothreitol (Sigma-Aldrich Corp.) or 0.1% DMSO were used as positive or negative controls, respectively. Cells were then incubated at 37°C with 5% CO_2_ overnight. After 24 h, diamide (Sigma-Aldrich Corp.) was added to all wells at a final concentration of 125 µM from a 100 mm stock solution prepared in DMSO and allowed to incubate at 37°C with 5% CO_2_ overnight (14–18 h). Plates were then washed with PBS, supplemented with 1 µM Calcein-AM (Molecular Probes, Invitrogen Corp.) and incubated at room temperature for 45 min. Cells were again washed with PBS to remove residual dye, and read on BMG PolarStar Optima with 485 excitation and 520 emission wavelengths (BMG LabTech).

### Western blot analysis

Cells were lysed using lysis buffer (Promega Corp.) supplemented with a complete protease inhibitor cocktail (Roche Applied Science) and phenylmethylsulfonyl fluoride (Sigma-Aldrich Corp.). Tissues were further homogenized using 0.5 mm glass beads in a Bullet Blender high-throughput homogenizer (Next Advance, Inc.). After pelleting cellular debris by spinning at 16 000 rpm at 4°C for 15 min, protein was quantified by Bradford assay (66). For western blotting, 40–50 µg protein was added per lane of 4–12% Bis-Tris gels (Invitrogen Corp.). Primary antibodies were diluted in Odyssey blocking buffer (LI-COR Biosciences). Antibodies used included: anti-FXN (provided by Franco Taroni M.D., Istituto Besta), anti-HO1 (# sc-10789, Santa Cruz Biotechnology, Inc.), anti-NQO1 (#3187, Cell Signaling Technologies), anti-Gpx4 (#ab125066, AbCam), anti-nrf2 (sc-25820, Santa Cruz) and anti-actin (#A2668, Sigma). Direct conjugated secondary antibodies (anti-rabbit IRdye800Cw and anti-mouse IRdye680 from LI-COR) were used to detect and quantify the signal of primary antibodies and imaged using a LI-COR Odyssey.

### Bioinformatics and phylogenetic analysis of FXN locus

A position weight matrix was used to identify ARE sequences in FXN based on previously described functional ARE sequences (28). A pre-filter was used with the consensus sequence of nnnnnnTGACnnnGCnnnnnn to identify potential ARE sites. The position weight matrix was used to calculate the score for each of the pre-filtered hits. Phylogenetic footprinting was then done using Clustalw2, and a multiple alignment was done between the great apes (Homo sapiens, Pan troglodytes, Gorilla and Pongo abeli) in the region upstream of the FXN gene (29).

### Chromatin immunoprecipitation

Patient lymphoblasts were seeded at 0.5 × 10^6^/ml in tissue culture flasks, and incubated with 0.1% DMSO, 5 µM dyclonine or 5 µM dimethyl-fumarate. About ∼10 × 10^6^ cells were used for each condition. After 24 h, cells were treated with 18.5% paraformaldehyde to crosslink proteins to DNA. ChIP was performed using an EZ-Chip kit (EMD Millipore). After shearing by sonication, each cell lysate was split and incubated with either Anti-Nrf2 (#H-300) or anti-mouse IgG negative control (Santa Cruz Biotechnology, Inc.) and protein-G-conjugated agarose beads to immunoprecipitate cross-linked protein/DNA. Crosslinks were reversed by incubating at 65°C overnight, and DNA was purified with provided spin columns and buffers. DNA concentration was determined using a Nanodrop 2000c (Thermo Scientific Corp.), and then analyzed by quantitative real-time PCR.

### Quantitative real-time PCR

Quantitative PCR was performed using the Superscript III One Step kit (Invitrogen Corp) in a Roche Lightcycler 480 (Roche Diagnostics). Standard curves were generated for each primer set, and samples ﬁtted to the linear portion of the curve. Additionally, PCR products were run on DNA gels to verify single products. Primer sequences used were described previously and were as follows: FXNFWD 5′- ATCTTCTCCATCCAGTGGACCT-3′ and FXNREV 5′- GCTGGGCATCAAGCATCTTTT-3′; ARE16223FWD 5′- CCTGCCGTACTCAGTCCTTC-3′ and ARE16223REV 5′- CCACTCGGCTGTACTGTCTG-3′; NQO1FWD 5′-CCCTTTTAGCCTTGGCACGAAA-3′ and NQO1REV 5′- TGCACCCAGGGAAGTGTGTTGTAT-3′; HO1FWD 5′ CCCTGCTGAGTAATCCTTTCCCGA-3′ and HO1REV 5′- ATGTCCCGACTCCAGACTCCA-3′ (67).

### ARE reporter assay

HeLa cells were treated with Cignal Lenti Reporter ARE-luciferase lentiviral reagent (SABiosciences, Cat # CLS-2020L) and underwent selection with 1 µg/ml of puromycin to generate stable cell line. Hela-ARE stable cells were washed with PBS and 1× EDTA, and plated in phenol-free DMEM media supplemented with 10% FBS in white wall/bottom 96-well plates in 90 µl. Plates were incubated at 37°C for 2 h to allow cells to adhere. Drugs or vehicle were added to all wells (10 µl/well). Cell plates were incubated at 37°C for 24 h. About 75 µl/well of cell lysis/luciferase Bright-Glo reagent (Promega) were then added to all wells. Wells were mixed with a biohit electronic pipettor (100 µl 2×). After 5 min without shaking, luminescence was read on BMG Polarstar.

### Aconitase assay

Aconitase activity was measured in patient lymphoblast and mouse model cerebellar lysate using the Aconitase Assay Kit (Cayman chemicals, Cat # 700600) and performed according to the manufacturer's instructions.

### Succinate dehydrogenase assay

Succinate dehydrogenase activity was measured (67) in mouse model liver lysate using the Complex II Enzyme Activity Microplate Assay Kit (Abcam, Cat # ab109908) and performed according to the manufacturer's instructions.

### Histone methyltransferase assay

G9 methyltransferase activity was measured in healthy lymphoblast nuclear extracts (Epiquik Nuclear Extraction Kit, Epigentek, Cat # OP-0002) and using the EpiQuik H3K9 Histone Methyltransferase Activity/Inhibition Assay Kit (Epigentek, Cat # P-3003) and performed according to the manufacturer's instructions.

### Animal procedures and behavioral assessments

All animal procedures were approved by the Institutional Animal Care and Use Committee at the University of California, Davis with adherence to the NIH Guide for the Care and Use of Laboratory Animals. The age of mice used for all studies was between 6 and 14 months and was age-matched within experiments. Drug dosing was done i.p. formulated in a DMSO and PBS mixture, and p.o. formulated in a DMSO, water and peanut butter mixture. Behavior testing was performed at the University of California, Davis Mouse Behavioral Analysis Laboratory. A colony of YG8R B6.Cg-Fxn^tm1Mkn^ Tg(FXN)YG8Pook/J mice was established at UC Davis (Jackson Labs, # 012253) (68). For our testing, we used both hemizygous mice [FA-YG8 (*hFXN*^+/−^ with FXN (GAA)_190_ expansion; m*Fxn*^−/−^)] containing one allele of the mutant FXN transgene and one KO (and thus the least amount of FXN) and homozygous mice [FA-YG8 (*hFXN*^+/+^ with FXN (GAA)_190_ expansion; m*Fxn*^−/−^], containing two alleles of the transgene (and thus higher levels of FXN), and wild-type (WT) mice. In addition, the B6.Cg-Fxn^tm1Mkn^ Fxn^tm1Pand^/J strain was also established and used (Jackson Labs, # 014162), and is referred to as FA-PandKIKO mice [*mFxn*^+/−^ with FXN (GAA)_230_ expansion; m*Fxn*^−/−^] (69,70). Both of these mice are bred on a C57BL/6J background.

Level beam performance was measured by placing mice on a 100 cm long level beam with a line marked 10 cm from each end as the start and end lines. A 60 W desk lamp was used as an aversive stimulus at the starting end of the beam, and an enclosed shelter remained at the end of the beam. A training beam of 21 mm diameter was used for three training trials, with at least 10 min of rest allowed for each animal between beam crosses. Subsequently, three trials were conducted for 21, 16 and 9 mm wide beams. Time was recorded for each animal to cross from start to finish. Additionally, a video camera recorded every trial and was later processed for quantification of foot slips as errors. Experimenter who recorded times and errors was blinded to treatment groups.

### Clinical human buccal cell collection

Eight FA patients and two healthy volunteers were dosed with a 1% dyclonine oral rinse 2× daily for 30 s for 7 days. Samples were collected prior to and at the end of 1 week dosing. Buccal cells were collected from patients and healthy controls using MasterAmp Buccal Swab brushes (Epicentre, Illumina Corp.). Swabs were gently twirled against the inside of the right cheek for 30 s. The swab was then removed from the mouth and dipped into a tube containing 500 µl of ice cold extraction buffer (AbCam, #ab109877). The swab brush was placed gently in the tube of buffer for ∼10 s to dislodge the cells. Tubes were frozen at −20°C until all samples received so FXN measurement could be performed at one time.

### FXN immunoassay protocol

FXN expression in buccal cells was measured using a commercially dipstick immunoassay for FXN (AbCam, #ab109877) and mitochondrial complex IV (AbCam, #ab109881) expression according to the manufacturer's instructions. Buccal cells were lysed using extraction buffer supplied with the assay kit and cellular debris pelleted. Protein concentration was measured by Bradford assay. About 10 µg of peripheral blood mononucleated cell protein in 25 µl of extraction buffer was mixed with 25 µl of blocking buffer and added to individual wells on a 96-well plate with gold-conjugated mAb at the bottom of each well. Samples were measured as duplicates. After 5 min incubation at room temperature, dipsticks were added into the well and allowed transfer onto the membrane, where FXN or complex IV was immunocaptured. This capture was quantified on dried dipsticks using a Hamamatsu dipstick reader (AbCam, #MS1000), and raw mABS (milli-Absorbance) values for FXN were normalized to complex IV raw values.

### Statistical analysis

Data are presented as mean ± SD or SEM, and the significance of the difference between groups was evaluated with the Student's *t*-test (two-tailed) or Pearson's correlation coefficient. A *P*-value of <0.05 was considered significant. Curve fits were done using GraphPad prism nonlinear regression analysis with a variable slope. Additionally, a 95% confidence interval was used in bioinformatics analysis of ARE sites in FXN gene.

### Study approval

The human clinical study was reviewed and approved by the Investigational Review Board of the University of California, Los Angeles (proof of concept of dyclonine in buccal cells of FA patients, UCLA IRB#13-000478). All patients were provided written informed consent prior to initiation of the study. All animal studies and procedures were approved by the Institutional Animal Care and Use Committee at the University of California, Davis.

## SUPPLEMENTARY MATERIAL

Supplementary Material is available at *HMG* online.

## FUNDING

This work was supported by funding from the Friedreich's Ataxia Research Alliance, the National Institutes of Health (PHS NIH RO1 NS077777-15, PO1 AG025532 and R01 EY012245) and the NIGMS-funded Pharmacology Training Program (T32GM099608). Funding to pay the Open Access publication charges for this article was provided by the NIH.

## Supplementary Material

Supplementary Data

## References

[1] Campuzano V., Montermini L., Lutz Y., Cova L., Hindelang C., Jiralerspong S., Trottier Y., Kish S.J., Faucheux B., Trouillas P. (1997). Frataxin is reduced in Friedreich ataxia patients and is associated with mitochondrial membranes. Hum. Mol. Genet..

[2] Koeppen A.H., Michael S.C., Knutson M.D., Haile D.J., Qian J., Levi S., Santambrogio P., Garrick M.D., Lamarche J.B. (2007). The dentate nucleus in Friedreich's ataxia: the role of iron-responsive proteins. Acta Neuropathol. (Berl.).

[3] Koeppen A.H., Morral J.A., Davis A.N., Qian J., Petrocine S.V., Knutson M.D., Gibson W.M., Cusack M.J., Li D. (2009). The dorsal root ganglion in Friedreich's ataxia. Acta Neuropathol. (Berl.).

[4] Albano L.M.J., Nishioka S.A.D., Moysés R.L., Wagenführ J., Bertola D., Sugayama S.M.M., Chong A.K. (2002). Friedreich's ataxia: cardiac evaluation of 25 patients with clinical diagnosis and literature review. Arq. Bras. Cardiol..

[5] Dürr A., Cossee M., Agid Y., Campuzano V., Mignard C., Penet C., Mandel J.-L., Brice A., Koenig M. (1996). Clinical and genetic abnormalities in patients with Friedreich's ataxia. N. Engl. J. Med..

[6] Filla A., De Michele G., Cavalcanti F., Pianese L., Monticelli A., Campanella G., Cocozza S. (1996). The relationship between trinucleotide (GAA) repeat length and clinical features in Friedreich ataxia. Am. J. Hum. Genet..

[7] Lu C., Cortopassi G. (2007). Frataxin knockdown causes loss of cytoplasmic iron–sulfur cluster functions, redox alterations and induction of heme transcripts. Arch. Biochem. Biophys..

[8] Al-Mahdawi S., Pinto R.M., Varshney D., Lawrence L., Lowrie M.B., Hughes S., Webster Z., Blake J., Cooper J.M., King R. (2006). GAA repeat expansion mutation mouse models of Friedreich ataxia exhibit oxidative stress leading to progressive neuronal and cardiac pathology. Genomics.

[9] Condò I., Ventura N., Malisan F., Tomassini B., Testi R. (2006). A pool of extramitochondrial frataxin that promotes cell survival. J. Biol. Chem..

[10] Montermini L., Richeter A., Morgan K., Justice C.M., Julien D., Castellotti B., Mercier J., Poirier J., Capozzoli F., Bouchard J.P. (1997). Phenotypic variability in Friedreich ataxia: role of the associated GAA triplet repeat expansion. Ann. Neurol..

[11] De Biase I., Chutake Y.K., Rindler P.M., Bidichandani S.I. (2009). Epigenetic silencing in Friedreich ataxia is associated with depletion of CTCF (CCCTC-binding factor) and antisense transcription. PLoS ONE.

[12] Shan Y., Schoenfeld R.A., Hayashi G., Napoli E., Akiyama T., Iodi Carstens M., Carstens E.E., Pook M.A., Cortopassi G.A. (2013). Frataxin deficiency leads to defects in expression of antioxidants and Nrf2 expression in dorsal root ganglia of the Friedreich's ataxia YG8R mouse model. Antioxid Redox Signal.

[13] Shelmire B., Gastineau F., Shields T.L. (1955). Evaluation of a new topical anesthetic, dyclonine hydrochloride. Arch. Dermatol..

[14] Florestano H., Bahler M. (1956). Antimicrobial properties of dyclonine hydrochloride, a new topical anesthetic. J. Am. Pharm. Assoc..

[15] Sinha B., Pattabhi V., Nethaji M., Gabe E. (1987). Structure of a local anaesthetic: dyclonine hydrochloride. Acta Crystallogr C.

[16] Yang J., Cavadini P., Gellera C., Lonnerdal B., Taroni F., Cortopassi G. (1999). The Friedreich's ataxia mutation confers cellular sensitivity to oxidant stress which is rescued by chelators of iron and calcium and inhibitors of apoptosis. Hum. Mol. Genet..

[17] Chantrel-Groussard K., Geromel V., Puccio H., Koenig M., Munnich A., Rötig A., Rustin P. (2001). Disabled early recruitment of antioxidant defenses in Friedreich's ataxia. Hum. Mol. Genet..

[18] Paupe V., Dassa E.P., Goncalves S., Auchère F., Lönn M., Holmgren A., Rustin P. (2009). Impaired nuclear Nrf2 translocation undermines the oxidative stress response in Friedreich ataxia. PLoS ONE.

[19] Kosower N.S., Kosower E.M. (1995). Diamide: an oxidant probe for thiols. Methods Enzymol..

[20] Kosower N.S., Kosower E.M., Wertheim B., Correa W.S. (1969). Diamide, a new reagent for the intracellular oxidation of glutathione to the disulfide. Biochem. Biophys. Res. Commun..

[21] Liu L., Trimarchi J.R., Keefe D.L. (1999). Thiol oxidation-induced embryonic cell death in mice is prevented by the antioxidant dithiothreitol. Biol. Reprod..

[22] Verdon E., Couedor P., Sanders P. (2007). Multi-residue monitoring for the simultaneous determination of five nitrofurans (furazolidone, furaltadone, nitrofurazone, nitrofurantoine, nifursol) in poultry muscle tissue through the detection of their five major metabolites (AOZ, AMOZ, SEM, AHD, DNSAH) by liquid chromatography coupled to electrospray tandem mass spectrometry—in-house validation in line with Commission Decision 657/2002/EC. Anal. Chim. Acta.

[23] Sangster N., Rickard J., Hennessy D., Steel J., Collins G. (1991). Disposition of oxfendazole in goats and efficacy compared with sheep. Res. Vet. Sci..

[24] Riah O., Dousset J.-C., Courriere P., Stigliani J.-L., Baziard-Mouysset G., Belahsen Y. (1999). Evidence that nicotine acetylcholine receptors are not the main targets of cotinine toxicity. Toxicol. Lett..

[25] Kim S.-J., Park C., Han A.L., Youn M.-J., Lee J.-H., Kim Y., Kim E.-S., Kim H.-J., Kim J.-K., Lee H.-K. (2009). Ebselen attenuates cisplatin-induced ROS generation through Nrf2 activation in auditory cells. Hear. Res..

[26] Kensler T.W., Wakabayashi N., Biswal S. (2007). Cell survival responses to environmental stresses via the Keap1-Nrf2-ARE pathway. Annu. Rev. Pharmacol. Toxicol..

[27] Osburn W.O., Kensler T.W. (2008). Nrf2 signaling: an adaptive response pathway for protection against environmental toxic insults. Mutat Res.

[28] Wang X., Tomso D.J., Chorley B.N., Cho H.-Y., Cheung V.G., Kleeberger S.R., Bell D.A. (2007). Identification of polymorphic antioxidant response elements in the human genome. Hum. Mol. Genet..

[29] Larkin M., Blackshields G., Brown N., Chenna R., McGettigan P.A., McWilliam H., Valentin F., Wallace I.M., Wilm A., Lopez R. (2007). Clustal W and Clustal X version 2.0. Bioinformatics.

[30] Bidichandani S.I., Ashizawa T., Patel P.I. (1998). The GAA triplet-repeat expansion in Friedreich ataxia interferes with transcription and may be associated with an unusual DNA structure. Am. J. Hum. Genet..

[31] Sandi C., Pinto R.M., Al-Mahdawi S., Ezzatizadeh V., Barnes G., Jones S., Rusche J.R., Gottesfeld J.M., Pook M.A. (2011). Prolonged treatment with pimelic *o*-aminobenzamide HDAC inhibitors ameliorates the disease phenotype of a Friedreich ataxia mouse model. Neurobiol. Dis..

[32] Al-Mahdawi S., Pinto R.M., Ismail O., Varshney D., Lymperi S., Sandi C., Trabzuni D., Pook M. (2008). The Friedreich ataxia GAA repeat expansion mutation induces comparable epigenetic changes in human and transgenic mouse brain and heart tissues. Hum. Mol. Genet..

[33] Saveliev A., Everett C., Sharpe T., Webster Z., Festenstein R. (2003). DNA triplet repeats mediate heterochromatin-protein-1-sensitive variegated gene silencing. Nature.

[34] Greene E., Mahishi L., Entezam A., Kumari D., Usdin K. (2007). Repeat-induced epigenetic changes in intron 1 of the frataxin gene and its consequences in Friedreich ataxia. Nucleic Acids Res..

[35] Paredes-Gonzalez X., Fuentes F., Su Z.Y., Kong A.N. (2014). Apigenin reactivates Nrf2 anti-oxidative stress signaling in mouse skin epidermal JB6 P+ cells through epigenetics modifications. AAPS j..

[36] Zhang C., Su Z.-Y., Khor T.O., Shu L., Kong A.-N.T. (2013). Sulforaphane enhances Nrf2 expression in prostate cancer TRAMP C1 cells through epigenetic regulation. Biochem. Pharmacol.

[37] Tella S.R., Goldberg S.R. (1998). Monoamine transporter and sodium channel mechanisms in the rapid pressor response to cocaine. Pharmacol. Biochem. Behav..

[38] Catterall W.A., Mackie K. (1996). Local Anesthetics. *Goodman & Gilman*‘*s The Pharmacological Basis of Therapeutics*.

[39] Lai H.C., Jan L.Y. (2006). The distribution and targeting of neuronal voltage-gated ion channels. Nat. Rev. Neurosci..

[40] Bridwell-Rabb J., Winn A.M., Barondeau D.P. (2011). Structure–function analysis of Friedreich's ataxia mutants reveals determinants of frataxin binding and activation of the Fe–S assembly complex. Biochemistry.

[41] Shan Y., Napoli E., Cortopassi G. (2007). Mitochondrial frataxin interacts with ISD11 of the NFS1/ISCU complex and multiple mitochondrial chaperones. Hum. Mol. Genet..

[42] Tan G., Napoli E., Taroni F., Cortopassi G. (2003). Decreased expression of genes involved in sulfur amino acid metabolism in frataxin-deficient cells. Hum. Mol. Genet..

[43] Shan Y., Cortopassi G. (2012). HSC20 interacts with frataxin and is involved in iron–sulfur cluster biogenesis and iron homeostasis. Hum. Mol. Genet..

[44] Willis J.H., Isaya G., Gakh O., Capaldi R.A., Marusich M.F. (2008). Lateral-flow immunoassay for the frataxin protein in Friedreich's ataxia patients and carriers. Mol. Genet. Metab..

[45] Deutsch E.C., Santani A.B., Perlman S.L., Farmer J.M., Stolle C.A., Marusich M.F., Lynch D.R. (2010). A rapid, noninvasive immunoassay for frataxin: utility in assessment of Friedreich ataxia. Mol. Genet. Metab..

[46] Schmitz-Hübsch T., du Montcel S.T., Baliko L., Berciano J., Boesch S., Depondt C., Giunti P., Globas C., Infante J., Kang J.-S. (2006). Scale for the assessment and rating of ataxia development of a new clinical scale. Neurology.

[47] Fahey M., Corben L., Collins V., Churchyard A., Delatycki M. (2007). How is disease progress in Friedreich's ataxia best measured? A study of four rating scales. J. Neurol. Neurosurg. Psychiatry.

[48] Friedman L.S., Farmer J.M., Perlman S., Wilmot G., Gomez C.M., Bushara K.O., Mathews K.D., Subramony S., Ashizawa T., Balcer L.J. (2010). Measuring the rate of progression in Friedreich ataxia: implications for clinical trial design. Mov. Disord..

[49] Chutake Y.K., Costello W.N., Lam C., Bidichandani S.I. (2014). Altered nucleosome positioning at the transcription start site and deficient transcriptional initiation in Friedreich ataxia. J. Biol. Chem..

[50] Kim Y.-C., Masutani H., Yamaguchi Y., Itoh K., Yamamoto M., Yodoi J. (2001). Hemin-induced activation of the thioredoxin gene by Nrf2. A differential regulation of the antioxidant responsive element by a switch of its binding factors. J. Biol. Chem..

[51] Lu C., Schoenfeld R., Shan Y., Tsai H.-J., Hammock B., Cortopassi G. (2009). Frataxin deficiency induces Schwann cell inflammation and death. Biochim. Biophys. Acta.

[52] Hashemy S.I., Holmgren A. (2008). Regulation of the catalytic activity and structure of human thioredoxin 1 via oxidation and *S*-nitrosylation of cysteine residues. J. Biol. Chem..

[53] Cox A.G., Brown K.K., Arner E.S., Hampton M.B. (2008). The thioredoxin reductase inhibitor auranofin triggers apoptosis through a Bax/Bak-dependent process that involves peroxiredoxin 3 oxidation. Biochem. Pharmacol..

[54] Drechsel D.A., Patel M. (2010). Respiration-dependent H_2_O_2_ removal in brain mitochondria via the thioredoxin/peroxiredoxin system. J. Biol. Chem..

[55] Lopert P., Day B.J., Patel M. (2012). Thioredoxin reductase deficiency potentiates oxidative stress, mitochondrial dysfunction and cell death in dopaminergic cells. PLoS ONE.

[56] Weaver L.C., Richards A.B., Abreu B.E. (1960). Central nervous system effects of a local anesthetic dyclonine. Toxicol. Appl. Pharmacol..

[57] Selak M.A., Lyver E., Micklow E., Deutsch E.C., Önder Ö., Selamoglu N., Yager C., Knight S., Carroll M., Daldal F. (2011). Blood cells from Friedreich ataxia patients harbor frataxin deficiency without a loss of mitochondrial function. Mitochondrion.

[58] Huang Y., Khor T.O., Shu L., Saw C.L.-L., Wu T.-Y., Suh N., Yang C.S., Kong A.-N.T. (2012). A γ-tocopherol-rich mixture of tocopherols maintains Nrf2 expression in prostate tumors of TRAMP mice via epigenetic inhibition of CpG methylation. J. Nutr..

[59] Yu S., Khor T.O., Cheung K.-L., Li W., Wu T.-Y., Huang Y., Foster B.A., Kan Y.W., Kong A.-N. (2010). Nrf2 expression is regulated by epigenetic mechanisms in prostate cancer of TRAMP mice. PLoS ONE.

[60] Kalinin S., Polak P.E., Lin S.X., Braun D., Guizzetti M., Zhang X., Rubinstein I., Feinstein D.L. (2013). Dimethyl fumarate regulates histone deacetylase expression in astrocytes. J. Neuroimmunol..

[61] Frank H.Y., Catterall W.A. (2003). Overview of the voltage-gated sodium channel family. Genome Biol..

[62] Tan G., Chen L.-S., Lonnerdal B., Gellera C., Taroni F.A., Cortopassi G.A. (2001). Frataxin expression rescues mitochondrial dysfunctions in FRDA cells. Hum. Mol. Genet..

[63] Streck E.L., Czapski G.A., da Silva C.G. (2013). Neurodegeneration, mitochondrial dysfunction, and oxidative stress. Oxidative Med. Cell. Longevity.

[64] Subramaniam S.R., Chesselet M.-F. (2013). Mitochondrial dysfunction and oxidative stress in Parkinson's disease. Prog. Neurobiol..

[65] Wang X., Michaelis E.K. (2010). Selective neuronal vulnerability to oxidative stress in the brain. Front. Aging Neurosci..

[66] Kruger N.J. (1994). The Bradford method for protein quantitation. Basic protein and peptide protocols.

[67] Chorley B.N., Campbell M.R., Wang X., Karaca M., Sambandan D., Bangura F., Xue P., Pi J., Kleeberger S.R., Bell D.A. (2012). Identification of novel NRF2-regulated genes by ChIP-Seq: influence on retinoid X receptor alpha. Nucleic Acids Res..

[68] Al-Mahdawi S., Pinto R.M., Ruddle P., Carroll C., Webster Z., Pook M. (2004). GAA repeat instability in Friedreich ataxia YAC transgenic mice. Genomics.

[69] Cossée M., Puccio H., Gansmuller A., Koutnikova H., Dierich A., LeMeur M., Fischbeck K., Dollé P., Kœnig M. (2000). Inactivation of the Friedreich ataxia mouse gene leads to early embryonic lethality without iron accumulation. Hum. Mol. Genet..

[70] Miranda C.J., Santos M.M., Ohshima K., Smith J., Li L., Bunting M., Cossée M., Koenig M., Sequeiros J., Kaplan J. (2002). Frataxin knockin mouse. FEBS Lett..

