# Supplementary material for: Dyclonine rescues frataxin deficiency in animal models and buccal cells of patients with Friedreich's ataxia
Source: Hum Mol Genet. 2014 Aug 11;23(25):6848–62. doi: 10.1093/hmg/ddu408 (PMC4245046; doi:10.1093/hmg/ddu408)
Supplement: Supplementary Data [file supp_23_25_6848__index.html]

Dyclonine rescues frataxin deficiency in animal models and buccal cells of patients with Friedreich's ataxia — Dyclonine rescues frataxin deficiency in animal models and buccal cells of patients with Friedreich's ataxia — Supplementary Data 

# Dyclonine rescues frataxin deficiency in animal models and buccal cells of patients with Friedreich's ataxia

## Supplementary Data

Supplementary Data

**Files in this Data Supplement:**

- Supplementary Data - Doc file
