# Supplementary material for: Dyclonine rescues frataxin deficiency in animal models and buccal cells of patients with Friedreich's ataxia
Source: Hum Mol Genet. 2014 Aug 11;23(25):6848–62. doi: 10.1093/hmg/ddu408 (PMC4245046; doi:10.1093/hmg/ddu408)
Supplement: Supplementary Data [file supp_ddu408_ddu408supp.doc]

Supplementary Materials for

**Dyclonine rescues frataxin deficiency in animal models and buccal cells of patients with Friedreich’s Ataxia**

Sunil Sahdeo^1^, Brian Scott^1^, Marissa McMackin^1^, Mittal Jasoliya^1^, Brandon Brown^2^, Heike Wulff^2^, Sue Perlman^3^, Mark Pook^4^, Gino Cortopassi^1^

**This file contains:**

Materials and Methods

Figure S1. 2% agarose gel electrophoresis of PCR products after frataxin knockdown.

Figure S2. Dyclonine induces frataxin protein in mouse heart and liver *in vivo.*

Figure S3. Map of the region upstream of the frataxin gene showing potential ARE sites.

Figure S4. Effects of dyclonine on sodium channel.

Figure S5. Effects of dyclonine on *in vivo* hot plate response.

Figure S6. Dyclonine improves behavioral defects of FA mice.

Table S1. Summary of drugs that protect FA patient fibroblasts from diamide stress, induce ARE-luciferase activity and increase frataxin protein expression.

Table S2. Four week dosing with dyclonine or vehicle does not affect mouse body weight.

**Fig S1.** **2% agarose gel electrophoresis of PCR products after frataxin knockdown.** PCR products derived from primer sets for frataxin or GAPDH were separated using a 2% agarose gel in TAE buffer from 50B11 cells treated with SiRNA directed at frataxin or AllStars non-targeting siRNA negative control. Lane 1: GAPDH primer, AllStars transfection. Lane 2: GAPDH primer, SiFXN transfection. Lane 3: FXN, AllStars transfection. Lane 4: FXN primer, SiFXN transfection. This verifies frataxin knockdown in 50B11 cells shown in Fig. 1A-B.

**Fig S2.** **Dyclonine induces frataxin protein in mouse heart and liver *in vivo.*** To determine ability of dyclonine to reverse the *in vivo* FXN protein defect in heart and liver, the YG8 FA transgenic mice were dosed daily orally with 1 mg/kg dyclonine for 7 days. At the end of the study, the animals were sacrificed, and processed for biochemical analysis, i.e. Western blots of dyclonine treated and vehicle groups of liver and heart lysate frataxin level. Western blot densitometry analysis normalized to GAPDH (heart) or b-actin (liver) shows dyclonine induces FXN expression *in vivo* by 1.5 – 2 fold.

**Fig S3.** **Map of the region upstream of the frataxin gene showing potential ARE sites**. The top three candidates selected from position weight matrix are between 5000 -16000 bp upstream of the transcription start site. Arrows indicate top 3 candidate locations 4.9 kb, 5.6 kb and 16.7 kb upstream of the transcription start site for frataxin, with ARE scores of 12.9, 12.5 and 8.0. Location of GAA trinucleotide repeats is shown in purple.

**Fig. S4.** **Effects of dyclonine on sodium channel.** NaV1.2 inhibition is shown in whole cell patch clamp configuration at dyclonine concentrations from 0.5-100 µM in N1E-115 neuroblastoma cells.. IC_50_ = 5.76 ± 4.6 µM (4 cells per concentration). Pipette solution: KF, Bath: Na Ringer. Pulse protocol: 10 ms at -80, 10 ms at 0, 5 ms at -90.

**Fig S5.** **Effects of dyclonine on *in vivo* hot plate response.** Following intraperitoneal dosing with a single dose of 3 mg/kg dyclonine, there is no significant delay of response to hot plate test (p-values>0.3 for all timepoints). (Positive control is 10 mg/kg indomethacin; negative control mice are injected with vehicle, 5 mice per group)

**Fig S6. Dyclonine improves behavioral defects of FA mice. (A)** Dyclonine decreases latency to cross a level beam *in vivo*. FA-PandKIKO mice showed increase in beam crossing time compared to WT controls at 13 months of age. FA-PandKIKO mice were dosed with 25 mg/kg dyclonine or vehicle for four weeks *p.o.* Latency to cross a 16mm beam was the recorded time from start to finish of the beam, with the experimenter blinded to treatment groups. The plotted data represent the un-normalized mean latency to cross a beam post-dosing or pre-dosing. The apparent decreases in latency comparing post-vehicle and post-dyclonine treated FA-PandKIKO mice was not significant. Comparing post-vehicle treated FA-PandKIKO and WT mice was significant.  **(B)** Dyclonine decreases number of mouse errors across level beam *in vivo*. FA-PandKIKO mice showed pronounced increase in errors (footslips) while crossing a 16 mm width level beam compared to WT controls. FA-PandKIKO mice were treated with 25 mg/kg dyclonine or vehicle for four weeks *p.o.* Dyclonine appeared to decrease the number of errors made, though it was not significant. Errors made from start to finish of the beam were analyzed using video recordings of each mouse trial, with the experimenter blinded to treatment groups. The plotted data represent the mean number of errors made for each individual mouse after three trials. Positive controls were vehicle treated WT C57/Bl6 mice. **P* < 0.05, *t* test. (*n=7-9 mice per group, three trials per mouse*).

**Table S1.** **Summary of drugs that protect FA patient fibroblasts from diamide stress, induce ARE-luciferase activity and increase frataxin protein expression.** FA fibroblasts were pre-treated with 10 µM test compound, DMSO (neg. control), or 300 µM DTT (pos. control) for 24 hours and followed by 100 µM diamide for 24 hours. Cell viability was measured with Calcein AM. 1600 compounds were screened (diamide+drugs) for the ability to protect FA fibroblasts from diamide stress. Compounds that rescued from diamide toxicity greater than Mean + 2xSD advanced to secondary screening, which included replication of protective effect in a concentration-dependent manner, 0.01–30 µM, *n=2-3*. 33 drugs protected dose-dependently from diamide stress. Data is presented as mean EC_50_ and max fold activity for fold change protection from diamide. As a potential mechanism for protection from diamide, all 33 drugs were tested for ability to affect ARE-luciferase activity in a HeLa reporter cell line. ARE- luciferase reporter assay results are shown as mean fold over DMSO control, plus SD. Compounds were tested at 10µM for 24 hours before cell lysis and measurement of ARE-luciferase activity.

3Frataxin western blot protein expression results are shown as mean fold over DMSO control, plus SD. 10µM, 48 hours. *: >2xSD from (-) control. Additionally, as another mechanism for protection from diamide, frataxin western blot protein expression was measured by western blot analysis for frataxin normalized to actin expression in FA lymphoblasts treated with 10µM drug for 48 hours. Results are shown as mean fold over DMSO control, plus SD. Frataxin *: P = < 0.05, t-test. Compounds highlighted in grey are significant inducers of both ARE and frataxin.

**Table S2.** **Four week dosing with dyclonine or vehicle does not affect mouse body weight**. Oral dyclonine or vehicle treatment for 4 weeks at 25 mg/kg resulted in minor changes in body weight. Negative control mice were vehicle treated FA PandMkn mice; Positive control were vehicle treated WT C57/Bl6 mice.
